# How much could health worker absenteeism impact health outcomes? A modeling study of malaria in Kenya

**DOI:** 10.1093/oodh/oqae031

**Published:** 2024-12-02

**Authors:** Amy Finnegan, Janet Muriuki, Olivia Velez

**Affiliations:** Data Science, IntraHealth Internationall, 6340 Quadrangle Drive, #150, Chapel Hill, NC 27517, USA; Duke Global Health Institute, 301 Trent Dr, Duke University, Durham, NC 27710, USA; Health Workforce Development, IntraHealth International, IntraHealth Kenya, 9 West, Parklands/Highridge Ring Road, Lower Kabete Rd, Nairobi, Kenya; Center for Digital Health, IntraHealth International, 6340 Quadrangle Drive, #150, Chapel Hill, NC 27517, USA

**Keywords:** malaria, human resources for health, digital health, Kenya, absenteeism

## Abstract

When health workers are not at their posts, health care does not happen. Health worker absenteeism in Kenya was 53.8% in 2018 according to the World Bank Service Delivery Indicators Survey. Absenteeism is especially impactful for treatment of malaria where delays in treatment can lead to deadly consequences especially among the most vulnerable. Human resources management and development strengthening interventions using digital tools like iHRIS, IntraHealth International’s open-source, human resource information system, can enable data-driven decision making to plan and budget for health workers and dynamically redistribute them. These promising approaches can reduce systemic absenteeism, but little is known how much impact reduced absenteeism can have on health outcomes. In this study, the Spectrum Malaria tool, a dynamic malaria transmission model developed by Avenir Health, was used to test three scenarios of reduced absenteeism (5, 10 and 15%) to quantify the potential impact of absenteeism on malaria cases and deaths averted and *Plasmodium falciparum (P. falciparum)* prevalence among children ages 2–9 years in Kenya between 2023 and 2030. A small, 5% increase in effective coverage of treatment of uncomplicated cases of malaria with artemisinin combination therapy could result in a 12% decrease in uncomplicated cases, a 15% reduction in severe cases, and a 13% reduction in deaths among the population and a 24% reduction in *P. falciparum* prevalence among children aged 2–9 years. Stemming health worker absenteeism is a critical intervention in the fight against malaria and digital tools like iHRIS for health workforce planning can help reduce absenteeism.

**RESUMEN:**

Cuando los prestadores de salud no están en sus puestos, el cuidado de la salud no ocurre. El ausentismo entre los prestadores de salud en Kenia fue de 53.8% en 2018, según las encuestas de los Indicadores de Prestación de Servicios (IPS) del Banco Mundial. Ausentismo como este entraña un impacto especialmente fuerte en el tratamiento de la malaria, donde la demora puede tener consecuencias letales, especialmente entre los más vulnerables. Intervenciones con herramientas digitales que fortalecen la gestión y desarrollo de recursos humanos, como iHRIS (el sistema de información de recursos humanos de código abierto de IntraHealth International), hacen posible tomar decisiones basadas en datos en torno a la planificación y presupuestación de la fuerza laboral de prestadores de salud y a cómo distribuirla de forma dinámica. Estos prometedores enfoques pueden reducir el ausentismo sistémico, pero se sabe poco acerca del tamaño del impacto que esta reducción puede tener en los resultados de salud. En este estudio usamos Spectrum-Malaria (un modelo dinámico de transmisión de la malaria, desarrollado por Avenir Health) como herramienta para examinar tres escenarios de ausentismo reducido (del 5, 10, y 15%) a fin de cuantificar el impacto potencial del ausentismo sobre el número de casos y fallecimientos evitados, y la prevalencia de *Plasmodium falciparum* (*P. falciparum*) en niños de 2–9 años de edad en Kenia entre 2023 y 2030. Un incremento pequeño, de 5%, en la cobertura efectiva de casos de malaria con tratamientos combinados con artemisinina (TCA), podría resultar en una reducción de 12% en el número de casos sin complicaciones, una reducción de 15% en los casos severos, y una reducción de 13% en el número de muertes entre la población, además de suponer una reducción de 24% en la prevalencia de *P. falciparum* en niños de 2–9 años de edad. Restringir el ausentismo de los prestadores de salud es una intervención crítica en la lucha contra la malaria, y el uso de herramientas digitales de planificación de la fuerza laboral, como iHRIS, puede ayudar a reducir este ausentismo.

**RESUMO:**

Quando os profissionais de saúde não estão nos seus postos, os cuidados de saúde não são prestados O absentismo dos profissionais de saúde no Quénia foi de 53,8% em 2018, de acordo com o Inquérito de Indicadores de Prestação de Serviços do Banco Mundial. O absentismo tem um impacto especial no tratamento da malária, onde os atrasos no tratamento podem ter consequências mortais, especialmente entre os mais vulneráveis. As intervenções de gestão de recursos humanos e de reforço do desenvolvimento que utilizam ferramentas digitais como o iHRIS, o sistema de informação de recursos humanos de fonte aberta da IntraHealth International, podem permitir a tomada de decisões baseadas em dados para planear e orçamentar os profissionais de saúde e redistribuí-los dinamicamente. Estas abordagens promissoras podem reduzir o absentismo sistémico, mas pouco se sabe sobre o impacto que a redução do absentismo pode ter nos resultados sanitários. Neste estudo, a ferramenta Spectrum Malaria, um modelo dinâmico de transmissão da malária desenvolvido pela Avenir Health, foi utilizada para testar três cenários de redução do absentismo (5, 10 e 15%), para quantificar o impacto potencial do absentismo nos casos de malária e mortes evitadas e na prevalência de *Plasmodium falciparum* (*p falciparum*) entre crianças dos 2 aos 9 anos de idade no Quénia entre 2023 e 2030. Um pequeno aumento de 5% na cobertura efectiva do tratamento de casos não complicados de malária com ACT poderia resultar numa diminuição de 12% nos casos não complicados, numa redução de 15% nos casos graves e numa redução de 13% nas mortes entre a população, bem como numa redução de 24% na prevalência de p falciparum entre crianças dos 2 aos 9 anos de idade. Travar o absentismo dos profissionais de saúde é uma intervenção fundamental na luta contra a malária e ferramentas digitais como o iHRIS para o planeamento da força de trabalho no sector da saúde podem ajudar a reduzir o absentismo.

**RÉSUMÉ:**

Lorsque les agents de santé ne sont pas à leur poste, les soins de santé ne sont pas dispensés. L’absentéisme des agents de santé au Kenya était de 53,8% en 2018, selon l’enquête sur les Indicateurs de prestation de services de la Banque mondiale. L’absentéisme a un impact particulièrement important pour le traitement du paludisme, où les retards dans le traitement peuvent avoir des conséquences mortelles, en particulier parmi les plus vulnérables. Les interventions de renforcement de la gestion et du développement des ressources humaines utilisant des outils numériques comme iHRIS, le système d’information sur les ressources humaines open source d’IntraHealth International, peuvent permettre une prise de décision fondée sur les données pour planifier et budgétiser la gestion des agents de santé et les redistribuer de manière dynamique. Ces approches prometteuses peuvent réduire l’absentéisme systémique, mais on sait peu de choses sur l’impact qu’une réduction de l’absentéisme peut avoir sur les résultats de santé. Dans cette étude, l’outil Spectrum Malaria, un modèle dynamique de transmission du paludisme développé par Avenir Health, a été utilisé pour tester trois scénarios de réduction de l’absentéisme (5, 10 et 15%) afin de quantifier l’impact potentiel de l’absentéisme sur les cas de paludisme et les décès évités. et sur la prévalence de *Plasmodium falciparum* (*P. falciparum*) chez les enfants âgés de 2 à 9 ans au Kenya entre 2023 et 2030. Une légère augmentation de 5% de la couverture efficace du traitement des cas de paludisme non compliqués par l’ACT pourrait entraîner une diminution de 12% de ceux-ci, une réduction de 15% des cas graves et une réduction de 13% des décès au sein de la population, ainsi qu’une réduction de 24% de la prévalence de P. falciparum chez les enfants âgés de 2 à 9 ans. Réduire l’absentéisme des agents de santé est une intervention essentielle dans la lutte contre le paludisme et les outils numériques comme iHRIS pour la planification des personnels de santé peuvent contribuer à réduire l’absentéisme.

## INTRODUCTION

Malaria is a deadly infectious disease, but it is preventable and curable. The largest reductions in deaths and morbidity come from the timely, effective management of uncomplicated cases with artemisinin combination therapy (ACT) [1–3]. Delays in treatment due to staff shortages, absenteeism and long waiting times can waste precious time treating malaria especially among women, children and those suffering from malnutrition who are more susceptible to rapid declines from malaria infection [4]. A recent modeling study estimated that if the COVID-19 pandemic reduced access to effective malaria treatment coverage in malaria endemic areas across Africa by 25%, it could result in a 4.1% increase in cases and a 26.3% increase in deaths, threatening gains against malaria over the last two decades [3].

**Figure 1:**
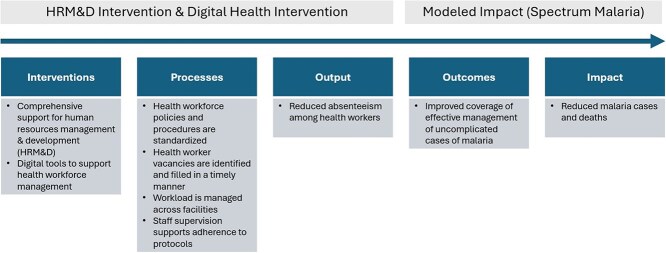
Impact model connecting digital health interventions for HRM&D to improved health outcomes

A strong, well-prepared health workforce is critical to delivering prompt and effective case management for malaria [5]. Insufficient numbers and a maldistribution of health workers in rural areas are a major challenge for primary health care service delivery, especially for malaria which requires prompt detection and treatment [4]. Staff shortages, absenteeism, long waiting times at public facilities and distrust in the quality of care have been documented as barriers to accessing care for malaria [5–7]. Staff absenteeism is a symptom of systemic problems in the management of the health workforce which can be addressed by comprehensive investments in strengthening human resources for health (HRH) units, improving the implementation of staff rewards and sanctions programs, timely compensation and increasing staff supportive supervision among other investments [8–10]. Figure 1 demonstrates the pathways through which digital health and human resources management and development (HRM&D) interventions can reduce absenteeism leading to increased effective management of uncomplicated cases of malaria with ACT and ultimately reduced cases and deaths.

The objective of this study is to extend the existing literature on health worker absenteeism to estimate the potential impact of absenteeism on malaria cases and deaths in Kenya between 2023 and 2030. The modeling approach will contribute to the literature on modeling the health impact of health systems strengthening and digital health interventions [11].

## STUDY SETTING

Kenya is a low- to middle- income country (LMIC) in East Africa with varying malaria endemicity throughout the country and by seasons and an absenteeism rate of 53.8% according to the 2018 World Bank Service Delivery Indicators Survey which is the most recent available data on absenteeism [12]. According to the 2020 Malaria Indicator Survey [13], 17% of children in Kenya had a fever in the 2 weeks before the survey. Of those with fever, 64% had care sought at all, with 68% of care sought in the public sector and 32% of care sought elsewhere (e.g. a private facility, drug shop or traditional healer). ACT is the gold standard treatment for malaria. For children with fevers who got care in the public sector, 71% received ACT. Only 9% of those who sought care outside the public sector received ACT. ACT care for malaria could be increased through more cases seeking care in the public sector where they are more likely to receive ACT; however, absenteeism can reduce the chances that care is sought in the public sector for illnesses by 35% and reduce the chance of receiving a malaria test at all by 28% [7]. Delayed care seeking, such as occurs when patients leave understaffed clinics and seek care elsewhere, can result in progression of disease leading to increased morbidity and mortality rates [4].

Between 2016 and 2021, IntraHealth International implemented the USAID-funded Human Resources for Health (HRH) Kenya project in 30 of 47 counties in Kenya. HRH Kenya’s goal was to strengthen HRM&D in the newly devolved county governments in Kenya including conducting evidence-based, data-driven health workforce planning and implementing digital human resources for health management using iHRIS, IntraHealth’s free and open-source health workforce planning tool. HRH Kenya supported counties were able to ensure timely renumeration of health workers, plan recruitments to replace retirees and other attritions, plan for budgeting and staffing gaps, and dynamically redeploy existing staff [14]. These interventions resulted in a reduction in absenteeism among health workers in counties exposed to HRH Kenya interventions compared to those not exposed by 12 percentage points (*P* < 0.28) and by 2 percentage points compared to the national level (*P* < 0.71) [8]. While the sample size was small and the results were not significant, the results are consistent with existing evidence that these types of compressive HRM&D interventions can reduce absenteeism [15–17] and improve overall service delivery outcomes [18]. However, no studies have modeled the potential magnitude of absenteeism’s impact on concrete, population-level health outcomes like malaria cases and deaths averted.

## DATA & METHODS

**Figure 2:**
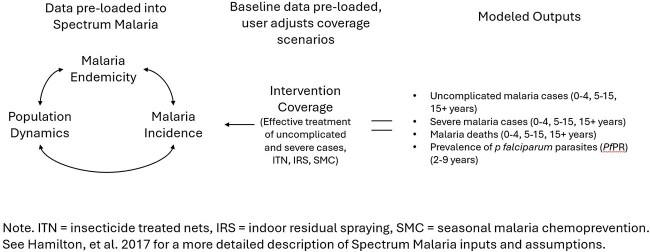
Simplified Spectrum Malaria model

The Spectrum Malaria module was used for this study. Spectrum Malaria, created by Avenir Health [2], is pre-loaded with a dynamic model of malaria transmission which considers malaria endemicity, malaria incidence and population dynamics to project the number of cases, deaths and prevalence of malaria in any given year at the county level in all malaria endemic countries [2]. Spectrum Malaria is designed to allow policymakers to model different levels of coverage of malaria interventions including effective management of uncomplicated malaria cases with ACT, effective management of severe cases, insecticide treated nets, indoor residual spraying (IRS) and seasonal chemoprophylaxis . The county-level values of intervention coverage and malaria endemicity and incidence come from the 2020 Malaria Atlas Project and population dynamics come from the DemProj and AIDS Incidence Model projections in Spectrum. Figure 2 shows a simplified version of the Spectrum Malaria model’s dynamic calculation methods which are described in detail elsewhere [2].

The purpose of this study was to understand how reductions in absenteeism through the use of digital tools and improved HRM&D management and thus increases in the effective treatment of uncomplicated cases of malaria with ACT could impact malaria cases, deaths and the prevalence of malaria parasites under three plausible absenteeism scenarios. Few previous studies have estimated the impact of absenteeism on malaria treatment. Zhang et al. [7] found an insignificant reduction of 14% (*P* < 0.22) in malaria treatment with an antimalaria drug due to no health workers being present during an illness episode in Uganda. While another study in Kenya found that HRM&D interventions could reduce absenteeism by between 2 and 12% (though the results were insignificant) [8]. Therefore, this study tested three plausible absenteeism scenarios: small (5%), modest (10%) and large (15%) increases in coverage of uncomplicated malaria cases with ACT due to decreases in absenteeism of the same magnitude.

We applied these increases in coverage uniformly across all 47 counties in Kenya in this hypothetical modeling study. The study used county level values of effective coverage of uncomplicated malaria cases with ACT for this analysis so that all counties would have the same percentage point increase in coverage; however, in practice absenteeism can differ by region, country and health facility. All coverage scenario values input into the model can be found in the Supplemental Appendix. Spectrum Malaria uses receipt of ACT by a child under five during the last malaria episode as the definition of effective coverage with ACT; ACT could come from any source. Spectrum Malaria models malaria transmission at the county level so this approach accounts for coverage while taking into account endemicity. In practice, a county may focus on increasing coverage in areas with higher endemicity. Cases and deaths from 2023 to 2030 were summed to get the total cases or deaths averted over the projected time period. The percent reduction from the baseline scenario was calculated for each of the scenarios tested.

## ETHICAL REVIEW

This study is based on modeled estimates and secondary data and therefore ethical review was not necessary.

## RESULTS

**Figure 3:**
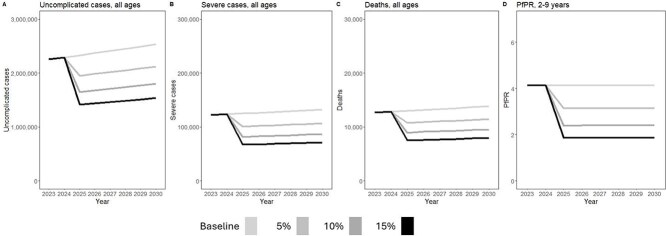
**Estimated outcomes over time by scenario (2023–2030).** Results for all ages are shown for uncomplicated cases (A), severe cases (B) and deaths (C). PfPR is shown for children aged 2–9 years (D).

**Figure 4:**
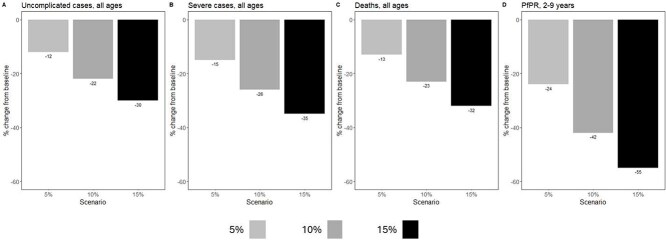
**Percent reduction in outcome for all ages compared to baseline scenario (2023–2030).** Results for all ages are shown for uncomplicated cases (A), severe cases (B) and deaths (C). PfPR is shown for children aged 2–9 years (D).

Each scenario of increased coverage of effective malaria treatment with ACT shows increasing impacts on reductions in uncomplicated cases, severe cases, deaths and the prevalence of malaria parasites among children aged 2–9. See Fig. 3 for projected trend analysis for each outcome and Fig. 4 for the percent reduction in cases, deaths and prevalence over the time period studied. Note that Spectrum Malaria is set up so that a change in coverage does not take effect until the following year which provides a conservative estimate of the impact of changes in intervention coverage on our outcomes of interest.

A decrease in absenteeism that results in a 5% (small) increase in coverage of uncomplicated cases of malaria with ACT (from 55.38 to 60.38% in this analysis) could result in a 12% reduction in uncomplicated cases, a 15% reduction in severe cases and 13% reduction in deaths among all age groups. The prevalence of *P. falciparum* parasites (PfPR) among children aged 2–9 years could be reduced by 24% in 2030 compared to the baseline in 2023. See Fig. 2 for full results. There is a similar pattern of reductions across age groups available in the Spectrum Malaria tool. See Table 1 for results.

**Table 1:** Scenario results

	**Baseline**		**Small (5%)**		**Modest (10%)**		**Large (15%)**	
	**Outcome**	**% change vs. baseline**	**Outcome**	**% change vs. baseline**	**Outcome**	**% change vs. baseline**	**Outcome**	**% change vs. baseline**
**All ages**								
Uncomplicated cases	19 133 726	0%	16 754 700	−12%	14 891 020	−22%	13 387 429	−30%
Severe cases	1 018 368	0%	868 035	−15%	751 160	−26%	660 411	−35%
Deaths	106 012	0%	92 156	−13%	81 138	−23%	71 913	−32%
**Age 0 to 4 years**								
Uncomplicated cases	5 162 331	0%	4 484 201	−13%	3 958 801	−23%	3 528 352	−32%
Severe cases	554 308	0%	475 976	−14%	415 351	−25%	366 972	−34%
Deaths	86 719	0%	75 007	−14%	65 755	−24%	58 015	−33%
**Age 5 to 15 years**								
Uncomplicated cases	4 951 113	0%	4 357 134	−12%	3 894 019	−21%	3 519 859	−29%
Severe cases	341 379	0%	288 709	−15%	247 769	−27%	216 762	−37%
Deaths	12 171	0%	10 867	−11%	9797	−20%	8895	−27%
**Age 15+ years**								
Uncomplicated cases	9 020 282	0%	7 913 365	−12%	7 038 200	−22%	6 339 218	−30%
Severe cases	122 681	0%	103 350	−16%	88 039	−28%	76 677	−37%
Deaths	7122	0%	6282	−12%	5586	−22%	5003	−30%
**Age 2–9 years**								
PfPR 2–9 years	4.14	0%	3.13	−24%	2.40	−42%	1.85	−55%

If reduced absenteeism resulted in a 10% (modest) increase in baseline coverage (from 55.38 to 65.38%, which is not an unreasonable increase because it falls within the 2–14% range suggested by Zhang et al. [7] and Finnegan et al. [8]), all age malaria uncomplicated cases could be reduced by 22%, severe cases could be reduced by 26% and deaths could be reduced by 23%. PfPR could be reduced by 42% among children aged 2–9 years in 2030 compared to the baseline year of 2023. See Table 1 for results.

In the unlikely scenario that coverage of effective treatment of uncomplicated cases with ACT could be increased by 15% (large effect) from reducing absenteeism alone, all age malaria uncomplicated cases, severe cases and deaths could be reduced by 30, 35 and 32%, respectively. PfPR could be reduced by 55% among children aged 2–9 years in 2030 compared to the baseline year of 2023. See Table 1 for results.

## DISCUSSION

A relatively small 5% increase in the effective treatment of uncomplicated cases of malaria with ACT in Kenya between 2023 and 2030 has the potential to avert 12% of uncomplicated cases, 15% of severe cases and 13% of deaths. The impact could be more substantial with more modest (10%) and large (15%) reductions in absenteeism. In Kenya, where absenteeism was recorded at 53.8% in 2018 [12], this scenario is not out of reach for the health system. Tools are available to reduce absenteeism like IntraHealth’s iHRIS planning software which is an open source global good [19] and HRH planning support which were associated with a 2–12% reduction in absenteeism in recent unpublished research [8, 14].

The results of this study using hypothetical scenarios are broadly similar with two recent studies of the effect of absenteeism on treatment for malaria in Uganda [7] and HIV testing during antenatal care in Kenya [20] as well as studies of other digital health interventions that can affect service delivery like digitized supply chain management [21]. Zhang et al. [7] find that there was no health worker present on 15% of days during the study in six districts in Eastern Uganda which resulted in 35% lower odds that someone would seek care in the public sector for a recent illness, 27% lower odds that malaria testing would be received and a 41% higher chance of paying out of pocket for malaria treatment. They did not estimate the impact of absenteeism on additional cases and deaths due to absenteeism. Goldstein et al. (2013) estimate that when nurses trained in prevention of mother to child transmission of HIV are absent when a mother makes her first antenatal care (ANC) visit to a facility in Kenya there is a 58 percentage point reduction in the likelihood that a mother will get an HIV test during her pregnancy and may contribute to 3.7 additional mother to child infections per 10 000 live births in Kenya and up to 14.6 additional infections per 10 000 live births across Africa if the absenteeism rate was 35% as suggested by prior studies [22]. Both studies use coarse measures of absenteeism, no health worker present during a visit to the facility, meaning these estimates could be overstated if one worker is missing and the other staff picks up the workload.

## LIMITATIONS

This study is not without limitations. The Spectrum Malaria [2] tool considers the effective treatment of uncomplicated cases to be the proportion of children with a fever in the 12 months before the study that received ACT, the first line treatment for malaria, from any source. Reductions in absenteeism in the public sector may not directly impact coverage especially when there is robust private sector availability of ACT. However, in Kenya where 68% of malaria cases sought care in the public sector, reducing public sector absenteeism likely drives the magnitude of the results. Additionally, the decision to seek care, where to seek it, the probability of obtaining ACT and the probability of being cured are complicated and complex processes. However, other studies that have used more complicated methods to approximate coverage [1, 3] reach the same general conclusion that malaria morbidity and mortality are driven by health workforce interventions rather than preventative measures like IRS or insecticide treated bed net distribution. Of course, effective treatment of uncomplicated cases with ACT can depend not just on a health worker being present to diagnose malaria and distribute ACT but on the availability of ACT in the supply chain, local health system dynamics, community behaviors and financial constraints for individuals when determining whether and where to seek care. Individuals could also get ACT from the informal sector when a public sector health worker is absent, though the quality of these drugs can be uncertain. If the most severe cases of malaria are likely to get ACT regardless of entry point into the health system, the number of deaths averted could be overestimated. Furthermore, holding all other interventions constant may not be the most likely comparative scenario for the time period under consideration since prevention and treatment interventions are all policy options that interact to provide reductions in malaria cases and deaths over time.

## CONCLUSION

Health workforce planning and the use of digital tools to manage the health workforce are critical to reducing staff shortages, long wait times and burnout among the health workforce which impact the coverage of interventions like malaria case management with ACT. Tools like iHRIS and wraparound HRM&D strengthening [8, 14, 18, 19] are promising interventions to support data-driven health workforce planning and policy formulation to ensure the right workforce quantity, skill mix, distribution and to help governments avert cases of malaria and deaths caused by absenteeism.

## Supplementary Material

ihris_absenteeism_and_malaria_in_Kenya-supplemental_appendix-accepted_oqae031

## Data Availability

The values used for this modeling study can be found in the Supplemental Appendix Table 1. They can be input into Avenir Health’s Spectrum Malaria tool.
