# Supplementary material for: How much could health worker absenteeism impact health outcomes? A modeling study of malaria in Kenya
Source: Oxf Open Digit Health. 2024 Dec 2;2(Suppl 2):ii18–24. doi: 10.1093/oodh/oqae031 (PMC11936326; doi:10.1093/oodh/oqae031)
Supplement: ihris_absenteeism_and_malaria_in_Kenya-supplemental_appendix-accepted_oqae031 [file ihris_absenteeism_and_malaria_in_Kenya-supplemental_appendix-accepted_oqae031.docx]

**Supplemental Appendix**

**How much could health worker absenteeism impact health outcomes? A modeling study of malaria in Kenya**

Amy Finnegan*^1,2^, Janet Muriuki^3^, Olivia Velez^4^

^1^Deputy Director, Data Science, IntraHealth International, Chapel Hill, North Carolina, USA

^2^Duke Global Health Institute, Duke University, Durham, North Carolina, USA

^3^Director, Health Workforce Development, IntraHealth International, IntraHealth Kenya, Nairobi, Kenya

^4^Chief Digital Health Officer, IntraHealth International, Chapel Hill, North Carolina, USA

*Corresponding author: Amy Finnegan, PhD, [afinnegan@intrahealth.org](mailto:afinnegan@intrahealth.org), 6340 Quadrangle Drive, #200, Chapel Hill, NC, USA 27517

Supplemental Table 1. County-level values of uncomplicated case coverage used in Spectrum Malaria scenarios.

| **Geography** | **2023 (baseline)** | **5%** | **10%** | **15%** |
| --- | --- | --- | --- | --- |
| National | 55.38 | 60.38 | 65.38 | 70.38 |
| Baringo | 48.24 | 53.24 | 58.24 | 63.24 |
| Bomet | 56.77 | 61.77 | 66.77 | 71.77 |
| Bungoma | 54.59 | 59.59 | 64.59 | 69.59 |
| Busia | 48.59 | 53.59 | 58.59 | 63.59 |
| Elgeyo-Marakwet | 51.16 | 56.16 | 61.16 | 66.16 |
| Embu | 48.67 | 53.67 | 58.67 | 63.67 |
| Garissa | 56.28 | 61.28 | 66.28 | 71.28 |
| Homa Bay | 56.15 | 61.15 | 66.15 | 71.15 |
| Isiolo | 48.50 | 53.50 | 58.50 | 63.50 |
| Kajiado | 55.54 | 60.54 | 65.54 | 70.54 |
| Kakamega | 48.60 | 53.60 | 58.60 | 63.60 |
| Kericho | 49.83 | 54.83 | 59.83 | 64.83 |
| Kiambu | 55.84 | 60.84 | 65.84 | 70.84 |
| Kilifi | 56.36 | 61.36 | 66.36 | 71.36 |
| Kirinyaga | 56.71 | 61.71 | 66.71 | 71.71 |
| Kisii | 55.27 | 60.27 | 65.27 | 70.27 |
| Kisumu | 55.44 | 60.44 | 65.44 | 70.44 |
| Kitui | 56.95 | 61.95 | 66.95 | 71.95 |
| Kwale | 56.79 | 61.79 | 66.79 | 71.79 |
| Laikipia | 56.22 | 61.22 | 66.22 | 71.22 |
| Lake Victoria | 52.26 | 57.26 | 62.26 | 67.26 |
| Lamu | 56.40 | 61.40 | 66.40 | 71.40 |
| Machakos | 53.36 | 58.36 | 63.36 | 68.36 |
| Makueni | 48.06 | 53.06 | 58.06 | 63.06 |
| Mandera | 54.62 | 59.62 | 64.62 | 69.62 |
| Marsabit | 55.96 | 60.96 | 65.96 | 70.96 |
| Meru | 56.37 | 61.37 | 66.37 | 71.37 |
| Migori | 51.59 | 56.59 | 61.59 | 66.59 |
| Mombasa | 46.96 | 51.96 | 56.96 | 61.96 |
| Murang'a | 54.91 | 59.91 | 64.91 | 69.91 |
| Nairobi | 56.30 | 61.30 | 66.30 | 71.30 |
| Nakuru | 56.42 | 61.42 | 66.42 | 71.42 |
| Nandi | 54.69 | 59.69 | 64.69 | 69.69 |
| Narok | 48.18 | 53.18 | 58.18 | 63.18 |
| Nyamira | 55.33 | 60.33 | 65.33 | 70.33 |
| Nyandarua | 49.09 | 54.09 | 59.09 | 64.09 |
| Nyeri | 56.81 | 61.81 | 66.81 | 71.81 |
| Samburu | 52.27 | 57.27 | 62.27 | 67.27 |
| Siaya | 56.53 | 61.53 | 66.53 | 71.53 |
| Taita Taveta | 49.28 | 54.28 | 59.28 | 64.28 |
| Tana River | 47.43 | 52.43 | 57.43 | 62.43 |
| Tharaka-Nithi | 47.99 | 52.99 | 57.99 | 62.99 |
| Trans Nzoia | 48.35 | 53.35 | 58.35 | 63.35 |
| Turkana | 51.06 | 56.06 | 61.06 | 66.06 |
| Uasin Gishu | 50.99 | 55.99 | 60.99 | 65.99 |
| Vihiga | 56.48 | 61.48 | 66.48 | 71.48 |
| Wajir | 55.94 | 60.94 | 65.94 | 70.94 |
| West Pokot | 55.94 | 60.94 | 65.94 | 70.94 |
